# Alleviating the public health burden of hypertension: debating precision prevention as a possible solution

**DOI:** 10.1080/16549716.2024.2422169

**Published:** 2024-11-11

**Authors:** Madeleine J. Samakosky, Shane A. Norris

**Affiliations:** a Department of Paediatrics, University of the Witwatersrand, Johannesburg, South Africa; b Department of Paediatrics, School of Clinical Medicine, University of the Witwatersrand, Johannesburg, South Africa; c Global Health Research Institute, School of Health and Human Development, University of Southampton, Southampton, UK; d SAMRC Developmental Pathways for Health Research Unit, Department of Paediatrics, School of Clinical Medicine, University of the Witwatersrand, Johannesburg, South Africa

**Keywords:** Precision prevention, hypertension, Africa, public health, cardiovascular disease (CVD), health interventions, epidemiology, sub-Saharan Africa, preventive medicine

## Abstract

Hypertension is a major global health concern, with deaths attributed to the condition expected to increase to 1.57 million by 2034, particularly affecting low-and-middle-income countries such as those within sub-Saharan Africa. Non-communicable diseases, with hypertension as a core contributor, account for 74.36% of global deaths. The burden of hypertension in sub-Saharan Africa is significant, with an estimated 10–20 million people currently affected. Systemic barriers, such as fragmented health services and socioeconomic inequalities, coupled with shifts in greater salt-intake, ultra-processed foods, more sedentary lifestyles, and overburdened healthcare services, have exacerbated elevated blood pressure and poorer management of people living with hypertension in sub-Saharan Africa. Most public health strategies focus on detecting, treating, and controlling hypertension through lifestyle modifications and medication. However, evidence suggests only 10% of population hypertension is well managed. This indicates a growing need to shift towards preventative efforts. Precision prevention, a tailored health intervention approach utilising individual and population-specific factors – genetic, environmental, and social determinants – offers a potential alternative. Precision prevention aims to deliver the right preventative measures to the right population at the right time, promising to enhance intervention efficiency and health outcomes. This paper highlights various intervention levers, including environmental, biological, and behavioural modifications, examines case studies from high-income countries, and discusses the potential for implementing precision prevention in South Africa. While precision prevention shows promise, we also discuss the significant barriers to its implementation in LMICs such as those within sub-Saharan Africa.

## Paper context

- **Main findings:** Hypertension affects 1 billion people globally and is a significant health burden in sub-Saharan Africa, where socioeconomic and lifestyle changes have increased its prevalence.
- **Added knowledge:** Precision prevention, a tailored health intervention approach, offers potential advantages over traditional methods by targeting individual and population-specific factors.
- **Global health impact for policy and action:** Implementing precision prevention strategies can improve health outcomes and reduce the burden of hypertension in sub-Saharan Africa, necessitating policy shifts and collaborative efforts to address infrastructural and resource challenges.

Hypertension poses a substantial global health challenge, with the number of deaths caused by the disease projected to rise to 1.57 million by 2034, especially in low- and middle-income countries (LMICs) [1]. Non-communicable diseases (NCDs), of which hypertension is a core contributor, account for 74.36% of global deaths [2]. Hypertension is a risk factor for cardiovascular disease (CVD) [3,4], a leading cause of death which contributes to approximately 54% of strokes and 47% of coronary heart diseases [5]. Even low-range pre-hypertension has been found to increase the risk of CVD, with estimates suggesting that eliminating prehypertension could prevent 15.9% of CVD cases, 14.6% of coronary heart diseases, and 19.6% of strokes [6]. Clinically, hypertension was estimated to have caused over 10 million preventable deaths annually, with LMICs suffering the most [7].

In South Africa, where hypertension affects over 40% of the population, awareness is alarmingly low [8]. Only 39.4% of hypertensive men and 53.8% of hypertensive women know their status, and a mere 10% have their blood pressure adequately controlled [8]. This lack of awareness and control exacerbates the public health burden, leading to increased rates of heart disease, stroke, and other non-communicable diseases. Financially, approximately 25% of South Africa’s healthcare expenditures are allocated to treating CVDs [9].

Urbanisation has led to significant lifestyle changes, including reduced physical activity and increased consumption of processed foods high in calories, sugar, salt, and unhealthy fats. With half the South African population being either overweight (23.6%) or obese (26.2%) [10], these dietary and lifestyle shifts contribute to higher rates of obesity and hypertension and result in subsequent systemic barriers to the effective detection, management, treatment, and prevention of hypertension [10,11]. Stress from rapid urbanisation, including crowded living conditions, job insecurity, and the pressures of urban life such as high living costs and limited access to affordable and convenient healthy foods, can also contribute to elevated blood pressure [12]. Additionally, economic disparities in South Africa result in unequal access to healthcare, healthy food, and opportunities for physical activity [13]. Lack of access to healthcare means that hypertension often goes undiagnosed and untreated until it reaches more severe stages. The healthcare infrastructure in South Africa is underdeveloped, with limited access to regular medical check-ups, hypertension screening, and long-term management. Shortages of healthcare professionals, particularly in rural areas, exacerbate the problem, making it difficult for people to get the care they need [13].

There are multiple levers that public health interventions can access to reduce hypertension risk and better manage hypertension (see Table 1). A successful preventative public health intervention is Finland’s North Karelia Project, which achieved significant reductions in coronary heart disease rates through coordinated public health campaigns [14]. As a result of this project, by 2011, CVD mortality among middle-aged men dropped from 690 to 100 per 100 000 [14]. This was achieved through the mobilisation of community organisations for health education, collaboration with non-governmental organisations to spread health messages, involving the private sector in reformulating food products (such as by reducing the salt content), and, critically, engaging with political leaders to influence policy changes [14]. While there are many levers utilised within this project, in particular, its success highlights the potential impact of political leadership and legislation changes.

**Table 1.** Levers of public health interventions.

Lever	Mechanism	Effectiveness	*South Africa **Ghana ***Africa (country not specified) ****Sub-Saharan Africa (country not specified) ******High-income country (country not specified) *******LMIC (country not specified)
**Category: Behavioural risk factor interventions**			
Policy and regulation	Legislation to reduce tobacco and alcohol consumption (e.g. taxes, restrictions on advertising, smoke-free zones)	***60% of tobacco policies were effective in reducing tobacco use; however, taxation policies had some nature of ineffectiveness due to the reasons that they are economic and regulative in nature that are easily manipulated by external factors like political will. [26]	
	Policies promoting healthy diets (e.g. sugar sweetened beverage taxes, regulations on trans fats, labelling requirements)	*The Health Promotion Levy (introduced by the South African government in 2018) led to a decline in sugar consumption from 519 mL/person per day to 443.39 mL/person per day. [27]	
	Urban design and transportation policies encouraging physical activity (e.g. promoting public transport, cycling and walking)	******The impact of urban form on active transport was expressed in 293 measurements, and 95.5% of these measurements were positively correlated with the use of walking and/or cycling for transport. [28]	
Public education	Mass media campaigns promoting healthy lifestyles	***Mass media campaigns and advertising bans were found to be the most effective policies for reducing tobacco use. [26]	
	Educational programs in schools, workplaces, and community centres on nutrition, physical activity, and smoking cessation	**A 6-week nutrition education intervention significantly improved children’s nutrition knowledge and attitudes, but not dietary diversity. [29]	
**Category: Biological risk factor interventions**			
Screening and treatment	Routine screening for high blood pressure, diabetes and high cholesterol	*Home-based hypertension screening reduced systolic blood pressure for women and younger men, but not for older men or diastolic blood pressure in either sex. [30]	
	Targeted treatment for high-risk individuals based on absolute risk assessments	****Well-designed risk triaging tools have the potential to improve health outcomes, but implementation will require commitment at the policy, operational, and funding levels. [31]	
Health care responses	Integrating NCD prevention into primary care services	*Integrating NCD care into primary services led to the control of 68% of hypertension, 82% of non-insulin dependent diabetes, and 84% of asthmas patients. [32]	
**Category: Environmental modifications**			
Food environment	Regulations or incentives for food manufacturers to reduce unhealthy ingredients (e.g. trans fats, excessive salt)	*Mandatory maximum sodium limits in processed foods led to a decrease in salt intake by 1.15 g per day. [16]	
	Fortification of foods with essential micronutrients (e.g. iodine, iron, Vitamin A)	*Food fortification with essential micronutrients has improved micronutrient intakes in 1- to 10-year old children, with the exception of folate in the Western Cape. [33]	
	Ensuring access to healthy foods (e.g. through subsidies or support for local markets)	*******Intensive agricultural interventions were associated with improved calorie, vitamin, fruit and vegetable intake. [34]	
Physical environment	Creating supportive environments for physical activity (e.g. parks, sports facilities)	*Currently, more than 50% of children meet physical activity recommendations. Access to activity-supportive environments is crucial for improving physical activity levels and nutrition in youth. [35]	
**Category: Economic and social policies**			
Economic incentives	Subsidies for healthy foods and physical activity programs	*Subsidies for healthy food purchases reduced regular fast-food consumption by 15% and increased fruit/vegetable consumption by 21%. [36]	
	Financial incentives for healthcare providers to focus on preventive care	*Incentivised health promotion programs, like the Vitality programme from Discovery Health, have shown a significant relationship between increased participation in wellness activities and lower healthcare expenditure. [37]	
Social support	Programs targeting social determinants of health (e.g. poverty reduction, education)	*Few/no effectiveness data*	
**Category: Community and social mobilisation**			
Community engagement	Involving community leaders and organisations in promoting health	*Community health workers promote health and link their communities to healthcare facilities despite resource limitations and lack of permanent employment. [38]	
	Establishing community-based programs for lifestyle changes	*Lifestyle Africa reduced haemoglobin A1c (HbA1c) for relatively little cost, with $71 in implementation cost per participant and a 0.26 improvement in HbA1c per participant. [39]	
**Category: Integrated and multisectoral approaches**			
Life course approach	Implementing interventions across different stages of life, from early childhood to old age	*Interventions targeting youth led to reductions in STI incidence and reported sexual or alcohol risk behaviours, with effects varying by intervention type. [40]	
Cross-sector collaboration	Coordinated efforts between health education, transportation and other sectors to create supportive environments for health	*Few/no effectiveness data*	

In South Africa, legislation was initiated to reduce salt in basic foods by setting the maximum allowed sodium levels in a wide range of processed foods and implementing public education campaigns to raise awareness about the risks of high salt intake [15]. While this policy has shown some positive results specifically in relation to reducing salt intake by 1.15 g per day [16,17], further research is needed to assess its full impact [18].

While traditional preventative public health interventions in LMICs have had some success, they often face several systemic constraints that limit their effectiveness. One major challenge is the availability and allocation of resources, including funding, trained personnel, and infrastructure, which can vary significantly across the regions [19]. Additionally, public health interventions must navigate complex regulatory environments and bureaucratic processes, which can delay implementation and reduce flexibility. Cultural and social barriers also play a significant role, as interventions may not be easily accepted or accessible to all populations due to differences in beliefs, health literacy, and socioeconomic status [20]. Finally, public health efforts can be constrained by competing health priorities and political pressures, which may divert attention and resources away from prevention-focused initiatives [19].

Precision prevention (PP) claims to overcome many of the constraints of traditional public health prevention interventions [21]. PP is a tailored health intervention approach that utilises individual and population-specific factors, including genetic, environmental, and social determinants, to deliver the right preventative measures to the right population at the right time [21–25]. By personalising interventions, optimising resource allocation and incorporating comprehensive data, PP has the potential to significantly improve health outcomes, particularly for NCDs such as hypertension in SSA [21,24]. PP offers a more tailored and iterative approach compared to traditional public health interventions. It employs individualised risk assessments, using biological and socio-environmental data to identify precise risks for each individual [22], rather than applying broad population-level strategies. PP integrates multi-dimensional data from genomic, environmental, behavioural, and social sources to compile a comprehensive health profile, enhancing the accuracy and precision of risk identification [21–23,25]. Traditional methods, however, often rely on aggregate data alone. Unlike the static, one-size-fits-all programmes of traditional public health, PP uses adaptive strategies allowing for real-time modifications of interventions based on evolving data. Lastly, PP focuses on addressing health disparities by tailoring interventions to marginalised or high-risk populations [25]. In contrast, traditional methods tend to apply uniform strategies across diverse groups. PPs unique approach holds potential to be more efficient in allocating resources, reducing costs, and maximising health outcomes in LMICs. PP has evolved from the field of precision medicine, which emerged in the early 2000s with the advent of genomic sequencing technologies. The evolution of PP is outlined in Table 2.

**Table 2.** Chronological diagram illustrating the evolution of PP.

Timeframe	Evolution of PP
Ancient Greek era (approx. 700 B.C.E)	Physicians select treatment based on individual characteristics, aiming for more effective healing. [48]
	The Corpus Hippocraticum states that “medicine is not absolute thus its directions cannot be generalised to everyone”. [48]
Traditional Chinese and Islamicate Medicine (up to 18^th^ century)	Physicians recognise that patients have unique balances of natural elements (humors or substances) and tailor treatments accordingly, demonstrating early forms of personalised care based on unique individual constitutions. [49]
Early 19^th^ century	The “comparative method” is promoted by people such as Pierre-Charles-Alexandre Louis. This involved dividing patients into groups, treating them differently and comparing outcomes. This laid the foundation for later precision medicine approaches. [50,51]
Late 19^th^ century	The relationship between genetics and disease are explored by Francis Galton and Karl Pearson. The use of biometry and eugenical reforms are promoted to influence health outcomes. [49,52]
	Raymond Pearl studies the interaction of “constitutional” (genetic) and environmental factors in disease distribution. [52]
Early 20^th^ century (1901–1918)	Inquiries into biochemical basis of large person-to-person variations in susceptibility to human-diseases and responses to medicines first begins. [53]
1985	In his seminal article entitled “Sick Individuals and Sick Populations” [54], Rose distinguishes a population approach as a primary goal of epidemiology: to seek control of the underlying causes of incidence.
1999	Francis Collins predicts that in the next 15–20 years, precision medicine will transform therapeutic medicine. [55]
2001	Genomic researchers, clinical programs, and professional journals widely adopt the language of precision medicine to frame their translational goals
2003	The Human Genome project is published. The main premise was that most human diseases have an underlying genetic component which can modulate disease onset, progression, presence of certain complications and treatment response. [56]
2011	The National Research Council’s “Toward Precision Medicine” [57] is published, positioning molecular biology at the heart of the ambition to develop a new taxonomy of disease.
2014	First use of the term “precision prevention” is published. [58]
2015	Francis Collins (Director of the National Institutes of Health) articulates the vision for precision medicine as the development of “prevention and treatment strategies that take individual variability into account”. [59]
	US Precision Medicine Initiative (recently rebranded the All of Us Research Program) is launched.
	The head of the Public Genomics Program at the US Centers for Disease Control and prevention writes: “While precision medicine is currently focused on treatment, a compelling case can be made for giving even more attention to early detection and disease prevention. Although personalised treatments can help save the lives of people who are already sick, disease prevention applies to all of us. ‘Precision prevention’ then may be useful in using both science and limited resources for targeting prevention strategies to subsets of the population.” [60]
2016	Adler & Prather (2015) publish “Risk for Type 2 Diabetes Mellitus: Person, Place and Precision Prevention”. [61]

While PP is an emerging approach in public health, it is not yet commonplace practice. However, PP is gaining traction, particularly with advances in data analytics, genomics, and digital health technologies, but its implementation is still in the early stages [41] In high-income countries, there are examples of the application of PP trials tackling cardiovascular health, hypertension, cancer, and Alzheimer’s disease [42–44]. A study by van den Brekel-Dijkstra et al. [45] evaluated the feasibility of a PP approach for CVDs. Conducted in Leidsche Rijn, Utrecht, the study involved inviting 800 residents aged 45–70 to complete a web-based health risk assessment. The assessment covered socio-demographic variables, family and personal medical history, lifestyle behaviours, and psychological factors to generate an individual cardiovascular risk profile. Participants received tailored web-based feedback and electronic referrals to local and online medical, psychological, and lifestyle interventions, with a follow-up questionnaire after six months. Out of 800 invited individuals, 29% (230) participated, and 39% (89) of these were identified as having an increased risk for CVD [45]. Among the high-risk group, 36% (32) underwent further biometric measures, revealing that 25% (8) had elevated blood pressure and 56% (18) had high total cholesterol levels [45]. One-third of participants initiated lifestyle changes, 20% planned future changes, 32% increased physical activity, and 28% adopted healthier eating habits [45]. While this study demonstrated the feasibility of effect of PP methodologies in primary care practice, its uptake was limited. This study demonstrates the feasibility of tailored interventions based on individual risk profiles, highlighting the potential for similar approaches in LMICs to target high-risk populations more effectively. By incorporating locally relevant socio-demographic, lifestyle, and medical factors, LMICs could adapt this model to address specific health challenges such as hypertension, while optimising resource allocation and improving public health outcomes.

The implementation of PP in LMICs is lagging. The absence of PP trials in LMICs can be attributed to several challenges, including limited healthcare infrastructure, the extensive data collection required, and significant cost barriers [46,47]. Despite these challenges, there are ongoing efforts to adapt and integrate PP strategies within LMICs, demonstrating a growing recognition of their potential benefits.

In South Africa, implementing PP interventions for hypertension could begin with the collection of individualised risk data such as biological data (e.g. genetic, proteomic, and metabolomic), as well as socioenvironmental data (such as lifestyle behaviours and demographic information) and environmental factors (such as access to healthy food). The development of robust data infrastructure would enable precise risk stratification, allowing healthcare researchers and practitioners to tailor interventions based on individual and community needs. Addressing the social determinants of health, such as income or education, would be central to these interventions. Partnerships among healthcare providers, researchers, government agencies, private companies, and community organisations would be critical to these interventions. It is important to emphasise that policy changes must be tailored to the specific needs of the population. For instance, South Africa’s salt reduction policy was introduced in response to the high prevalence of NCDs exacerbated by excessive salt intake. Similarly, any additional policy modifications should address the unique health challenges faced by South African communities, such as access to healthy food, health education, and socioeconomic disparities. Building on South Africa’s salt reduction policy, future policies should expand to incorporate PP strategies, focusing on individualised risk assessments and tailored interventions that account for biological, environmental, and socio-economic factors to more effectively address hypertension and related health disparities. Continuous data monitoring and iterative intervention design would be critical to the creation of effective, culturally relevant and sustainable PP interventions. These, as well as additional strategies that could be employed in future PP interventions in SA (as well as other LMICs) are detailed in Table 3.

**Table 3.** Strategies for PP interventions to be expanded in LMICs.

Strategy	Method	Detail	Evidence of application in LMICs * Indicates that they have been shown to be effective ** Indicates cost-effectiveness
Strengthening data infrastructure	Electronic Health Records	Developing and integrating electronic health record systems in LMICs can help track patient data over time, enabling the identification of high-risk individuals and communities.	Yes
	Population health data	Collecting and analysing data on population health trends, genetic predispositions, and environmental factors is crucial for developing targeted interventions.	Yes
	Genomic research	Expanding research into the genetic factors influencing hypertension in diverse populations can inform more precise strategies.	Yes
Leveraging mobile health (mHealth) technologies	Mobile applications	Utilising mobile apps to deliver personalised health advice, reminders for medication adherence, and lifestyle modification tips can reach a wide population in LMICs where mobile phone penetration is high.	Yes***
	SMS campaigns	Implementing SMS-based education and intervention programmes can help disseminate tailored information to individuals based on their risk factors and health profiles.	Yes* **
	Remote monitoring	Enabling remote blood pressure monitoring through mobile devices allows healthcare providers to track patients’ health and adjust interventions as needed.	Yes
Community-based interventions	Community health workers	Training community health workers to deliver personalised education and preventative care based on local population health data can bridge gaps in the formal healthcare system.	Yes***
	Culturally tailored programmes	Designing interventions that respect cultural practices and dietary habits can improve acceptance and adherence. This may include working with local leaders or using traditional communication methods.	Yes**
Policy and regulatory support	National guidelines	Developing and implementing national guidelines that incorporate PP approaches into primary healthcare can standardise best practices across regions.	Yes
	Affordable diagnostic tools	Promoting the use of cost-effective diagnostic tools and devices, like portable blood pressure monitors, can help in the early detection and continuous monitoring of hypertension.	Yes**
Public-private partnerships	Collaboration with tech companies	Partnering with tech companies to develop and distribute mHealth solutions that are affordable and accessible to low-income populations.	Yes
	Pharmaceutical partnerships	Working with pharmaceutical companies to ensure the availability of affordable antihypertensive medications that are tailored to the genetic profiles common in the region,	No
Education and awareness campaigns	Targeted education	Conducting public health campaigns that focus on high-risk populations identified through data analysis. These campaigns should educate people about the importance of monitoring blood pressure, reducing salt intake and other lifestyle changes.	Yes***
	School and workplace programs	Integrating hypertension prevention into school curricula and workplace wellness programs can reach people at various stages of life.	Yes***
Training and capacity building	Healthcare provide training	Provide training for the healthcare providers in LMICs on the principles and practices of PP can improve the quality of care.	Yes***
	Research and development	Encouraging local research into hypertension and its prevention can help build a knowledge base that is specific to the needs of the country or region.	Yes
Financial and logistical support	Subsidies and insurance	Developing health insurance schemes that cover preventive services and the costs associated with PP technologies can make these interventions more accessible.	Yes
	Funding for research	Increase funding for research on hypertension prevention in LMICs, particularly research that focuses on local contexts and needs.	Yes
Integration with existing healthcare programs	Combining with other health initiatives	Integrating hypertension prevention with existing healthcare programs, such as those for diabetes or maternal health, can create synergies and reduce costs.	Yes
	Routine screening	Incorporating blood pressure screening into routine health visits, immunisation programmes, or other community health activities can help identify at-risk individuals early.	Yes
Evaluation and feedback mechanisms	Monitoring outcomes	Establishing systems to monitor and evaluate the effectiveness of PP interventions allows for continuous improvement and scaling up of successful strategies.	No
	Patient feedback	Involving patients in the design and refinement of interventions ensures that the strategies are user-friendly and address real-world needs.	No

*While many of these methods have been applied in LMICs, they have not necessarily done so holistically as part of a PP intervention. Additionally, the application of these methods in a SSA context are particularly limited.

Implementing PP in SSA and other LMICs demands a balanced, multifaceted approach that combines innovation with pragmatism. Success hinges on overcoming systemic barriers by improving healthcare infrastructure and leveraging local expertise through extensive formative research to develop culturally appropriate strategies. By investing in scalable and adaptable solutions, fostering multi-sectoral and -disciplinary collaborations and strengthening healthcare infrastructure (e.g. by improving access to care), LMICs can begin to leverage the benefits of PP. It is essential to prioritise equitable access to these advancements and ensure that interventions are both cost-effective and culturally appropriate. Embracing these strategies has the potential to not only enhance the effectiveness of public health efforts but also to contribute to more sustainable and impactful health improvements across diverse populations.
